# Supplementary material for: TAD boundaries and gene activity are uncoupled
Source: bioRxiv. 2026 Feb 24:2025.12.13.694158. Preprint. [Version 3] doi: 10.64898/2025.12.13.694158 (PMC12724616; doi:10.64898/2025.12.13.694158)
Supplement: Supplement 1 [file NIHPP2025.12.13.694158v3-supplement-1.pdf]

bioRxiv preprint doi: https://doi.org/10.64898/2025.12.13.694158; this version posted February 25, 2026. The copyright holder for this preprint (which was not certified by peer review) is the author/funder. This article is a US Government work. It is not subject to copyright under 17 USC 105 and is also made available for use under a CC0 license.

SUPPLEMENTARY FIGURES

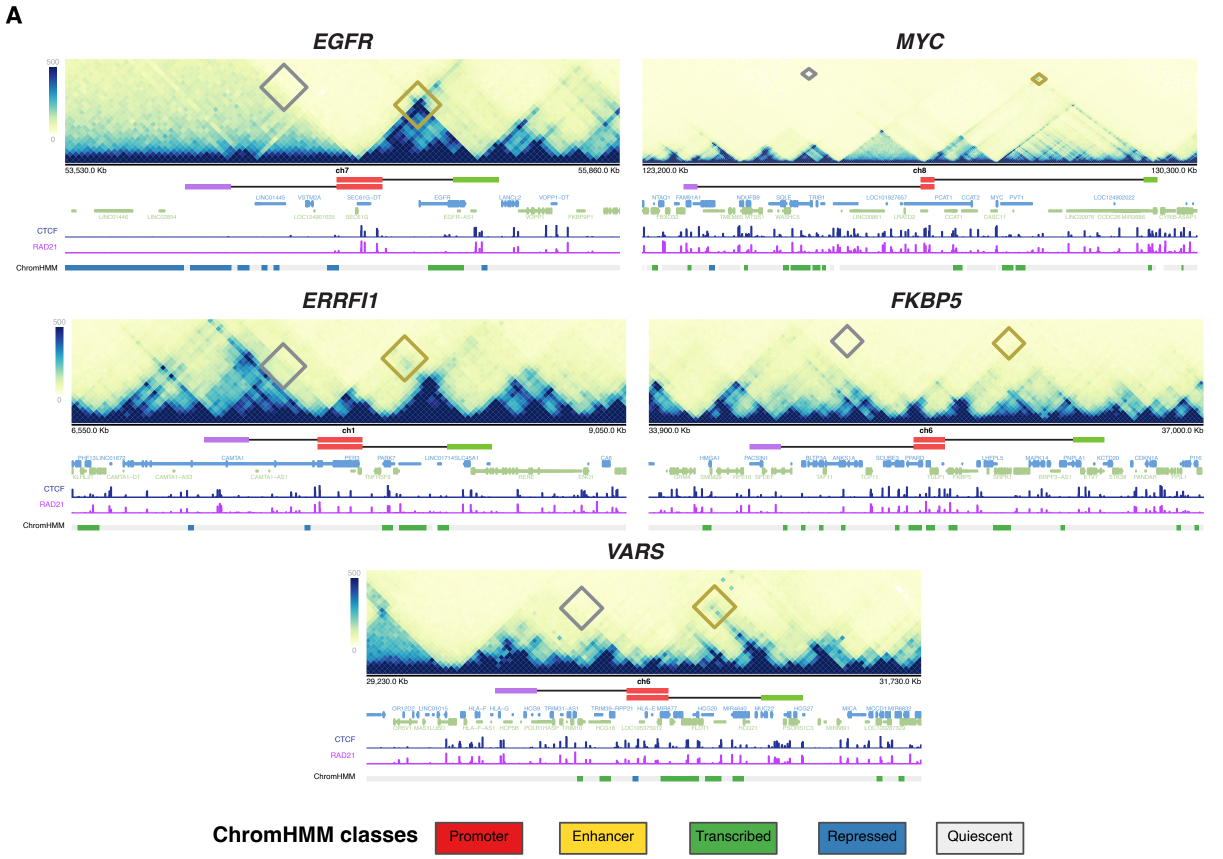

Supplementary Figure 1 Micro-C chromosome interaction maps and ChromHMM analysis of *EGFR*, *MYC*, *ERRFI1*, *FKBP5*, and *VARS2* TADs in HFFc6

ChromHMM chromatin states for two different foreskin fibroblast cell lines. The cytogenic chromosome band track (grey) indicates the chromosome location of the indicated loci. The gene reference track (blue) shows all coding and non-coding genes. Diamonds denote probe interaction sites in Micro-C for both the non-TAD probes (red) and the TAD boundary probes (blue).

bioRxiv preprint doi: https://doi.org/10.64898/2025.12.13.694158; this version posted February 25, 2026. The copyright holder for this preprint (which was not certified by peer review) is the author/funder. This article is a US Government work. It is not subject to copyright under 17 USC 105 and is also made available for use under a CC0 license.

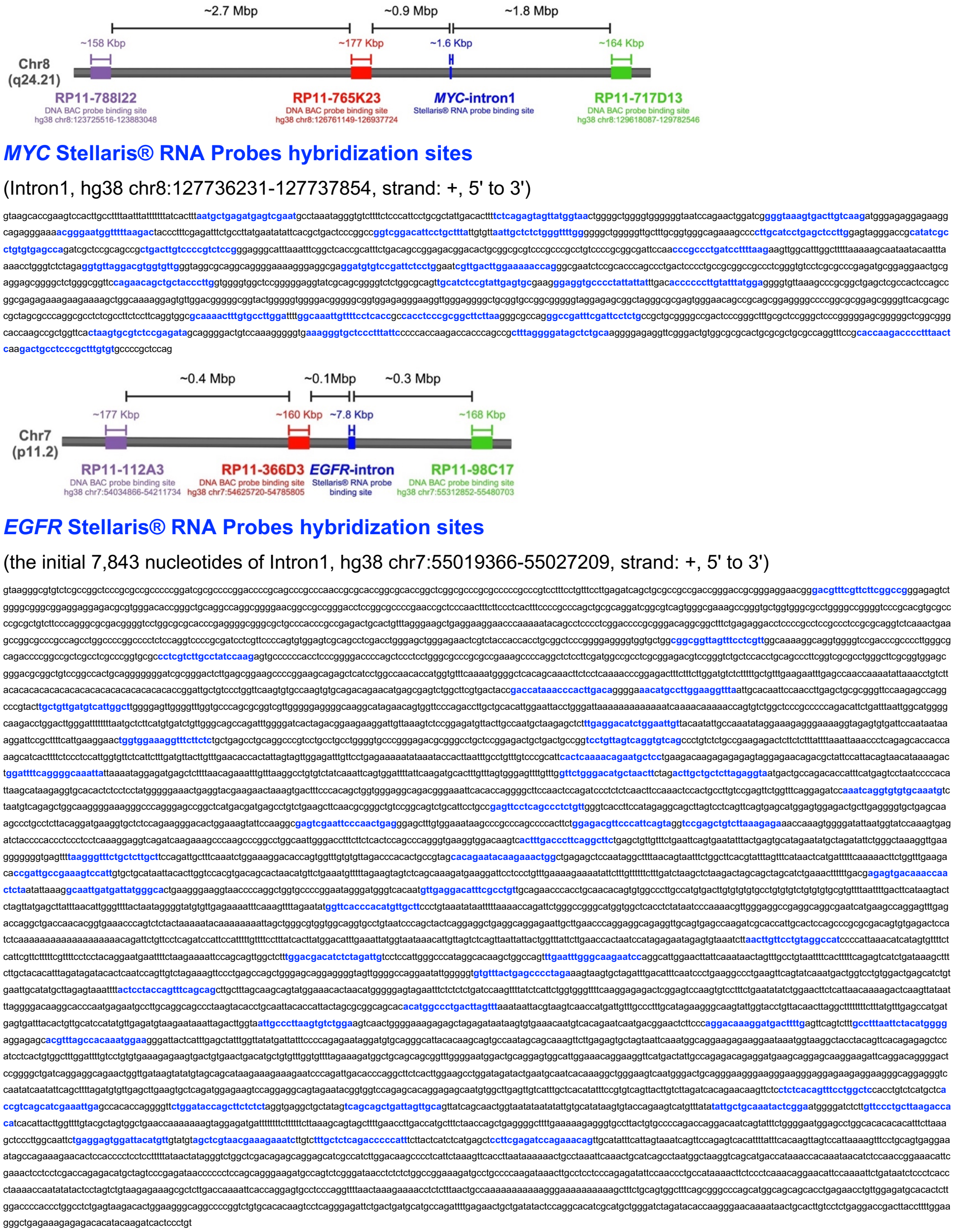

Supplementary Figure 2. Sequence and location of DNA and RNA probes binding sites for DNA/RNA HiFISH

Top: schematic representation of target regions. Oligonucleotide targeting sequences are indicated in blue. Bottom: target sequence regions.

bioRxiv preprint doi: https://doi.org/10.64898/2025.12.13.694158; this version posted February 25, 2026. The copyright holder for this preprint (which was not certified by peer review) is the author/funder. This article is a US Government work. It is not subject to copyright under 17 USC 105 and is also made available for use under a CC0 license.

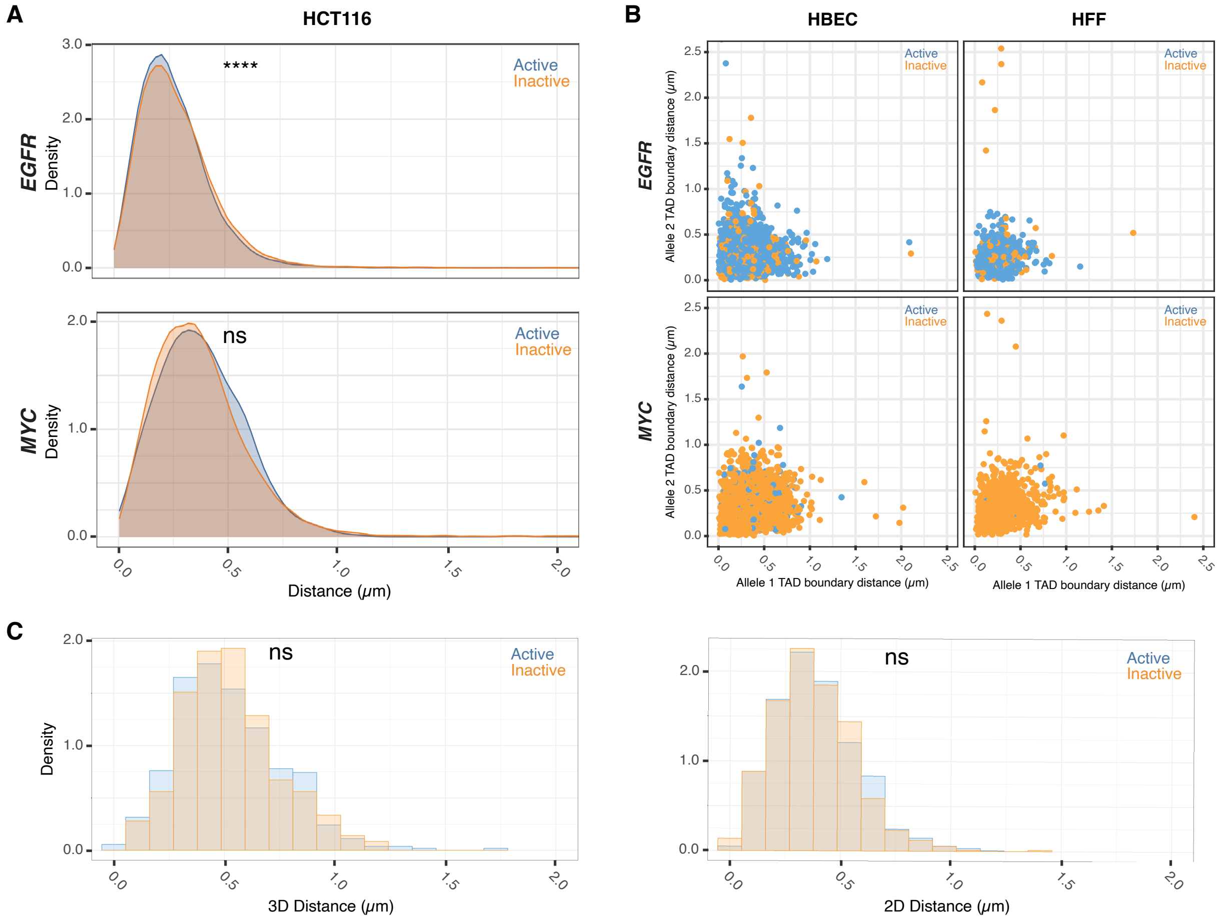

Supplementary Figure 3. TAD boundary proximity is uncoupled from allelic gene activity in single nuclei in both 2D and 3D imaging

(A) *Comparison of TAD boundary distances for EGFR and MYC alleles in HCT116 based on transcriptional activity status. Histograms of allele-specific distance distributions from a representative dataset from a single experiment. Mann–Whitney U test p-values are indicated as follows: ****p < 0.0001; ns, not significant (p ≥ 0.05).*

(B) *Comparative analysis of TAD boundary distances between active and inactive alleles within the same nucleus for EGFR and MYC loci in HBEC and HFF cells.*

(C) *Comparison of TAD boundary distances for MYC alleles in HBEC based on transcriptional activity status, measured using both 2D and 3D imaging. Histograms show allele-specific distance distributions from a representative dataset of a single experiment. Mann–Whitney U test p-values are indicated as follows: ns, not significant (p ≥ 0.05).*

bioRxiv preprint doi: https://doi.org/10.64898/2025.12.13.694158; this version posted February 25, 2026. The copyright holder for this preprint (which was not certified by peer review) is the author/funder. This article is a US Government work. It is not subject to copyright under 17 USC 105 and is also made available for use under a CC0 license.

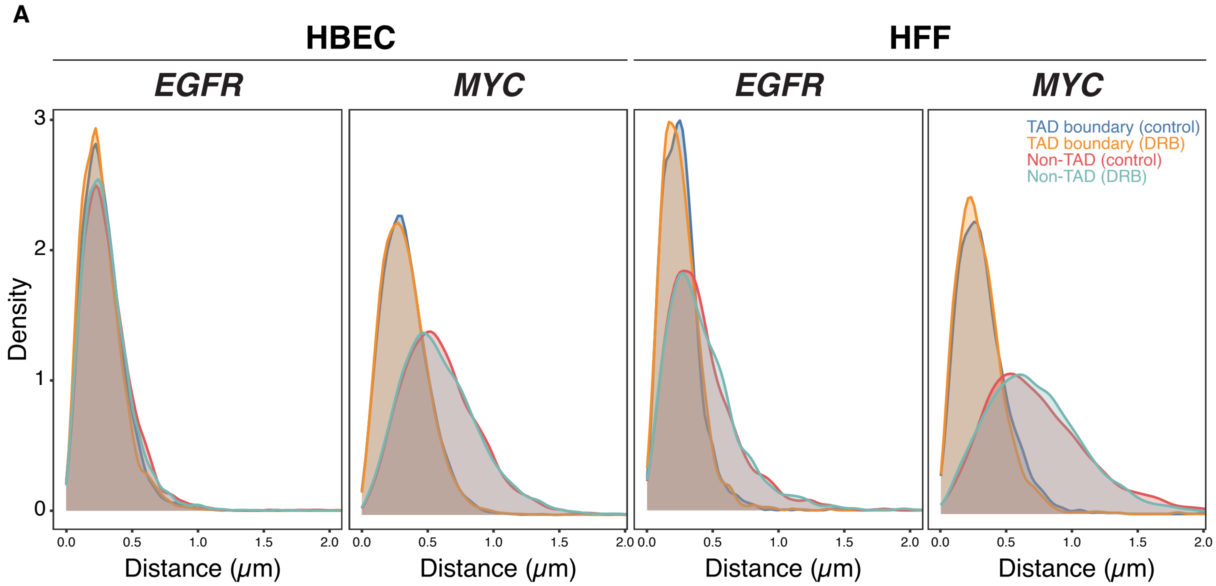

Supplementary Figure 4. Transcriptional inhibition does not affect the spatial organization of non-TAD control regions

(A) *Comparison of TAD boundary and non-TAD distances for EGFR and MYC alleles in HBEC and HFF based on transcriptional inhibition status. Histograms show allele-specific distance distributions from a representative dataset of a single experiment.*

bioRxiv preprint doi: https://doi.org/10.64898/2025.12.13.694158; this version posted February 25, 2026. The copyright holder for this preprint (which was not certified by peer review) is the author/funder. This article is a US Government work. It is not subject to copyright under 17 USC 105 and is also made available for use under a CC0 license.

A

Gene	2h Dex fold_change	4h Dex fold_change
EGFR	1.023666933	1.03900346
MYC	0.94073356	0.942055814
ERRFI1	4.21958045	3.046223714
FKBP5	3.601279644	8.110225204
VARS2	5.465222715	6.468733565

B

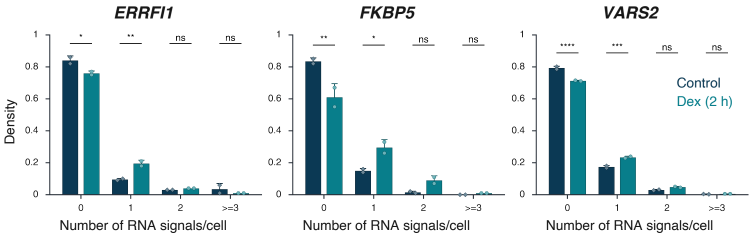

Supplementary Figure 5. RNA levels following DEX treatment

(A) *RNAseq analysis of ERRFI1, VARS2, and FKBP5 RNA levels following Dex treatment for the indicated durations in HBEC cells. Values were calculated RPKM fold-change (Dex/No_Dex) ratio. Data represent the mean of three independent experiments.*

(B) *Histograms of the distribution of nascent RNA transcription sites per nucleus in HBECs upon Dex treatment. Data represent values from at least two independent experiments (diamonds and circles); diamonds (EtOH control) and circles (Dex 2 h) represent the mean of means, and error bars indicate SD. P-values from two-way ANOVA with Bonferroni correction are shown as: ****p < 0.0001; ***p < 0.001; **p < 0.01; *p < 0.05; ns, not significant (p ≥ 0.05).*

bioRxiv preprint doi: https://doi.org/10.64898/2025.12.13.694158; this version posted February 25, 2026. The copyright holder for this preprint (which was not certified by peer review) is the author/funder. This article is a US Government work. It is not subject to copyright under 17 USC 105 and is also made available for use under a CC0 license.

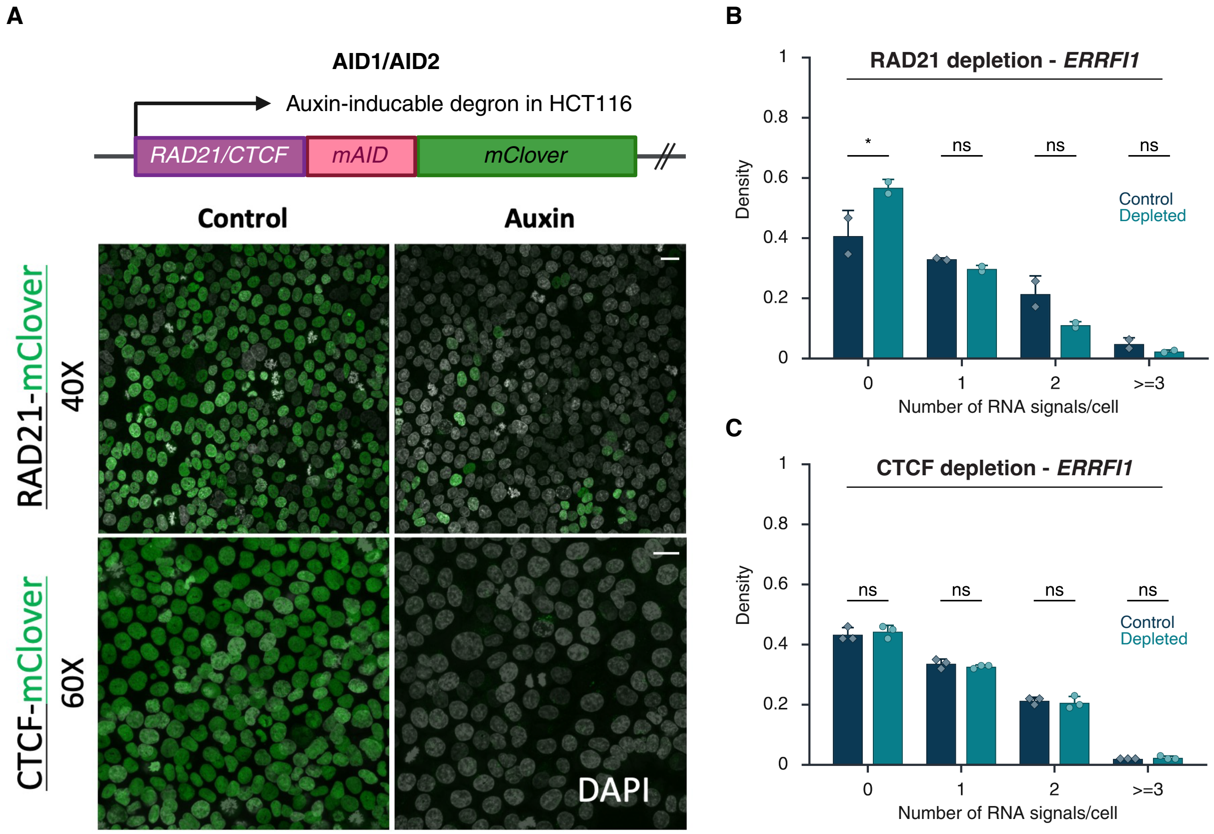

Supplementary Figure 6. Depletion of RAD21 and CTCF

(A) *Loss of RAD21 or CTCF in HCT116-RAD21-AID1 or HCT116-CTCF-AID2 cells, respectively, following DMSO (control) or auxin treatment. RAD21 and CTCF degradation were assessed using mClover- fluorescence (green). Scale bar: 20 µm*

(B-C) *Fraction of silent (0), monoallelic (1), biallelic (2), and triallelic or more (≥3) expression of the indicated genes in individual cells after 3 hours or no auxin treatment in HCT116-RAD21-AID1 (B) or HCT116-CTCF-AID2 (C) cells. Data represent values from at least two independent experiments (diamonds and circles); diamonds (DMSO control) and circles (RAD21 or CTCF-depleted) represent the mean of means, and error bars indicate SD. P-values from two-way ANOVA with Bonferroni correction are shown as: *p < 0.05; ns, not significant (p ≥ 0.05).*
